# MODB: a comprehensive mitochondrial genome database for Mollusca

**DOI:** 10.1093/database/baab056

**Published:** 2021-09-12

**Authors:** Jiangyong Qu, Yanran Xu, Yutong Cui, Sen Wu, Lijun Wang, Xiumei Liu, Zhikai Xing, Xiaoyu Guo, Shanshan Wang, Ruoran Li, Xiaoyue Sun, Xiang Li, Xiyue Wang, Tao Liu, Xumin Wang

**Affiliations:** College of Life Sciences, Yantai University, No.30 Qingquan Road, Laishan District, Yantai, Shandong 264005, China; College of Life Sciences, Yantai University, No.30 Qingquan Road, Laishan District, Yantai, Shandong 264005, China; College of Life Sciences, Yantai University, No.30 Qingquan Road, Laishan District, Yantai, Shandong 264005, China; College of Life Sciences, Yantai University, No.30 Qingquan Road, Laishan District, Yantai, Shandong 264005, China; College of Life Sciences, Yantai University, No.30 Qingquan Road, Laishan District, Yantai, Shandong 264005, China; College of Life Sciences, Yantai University, No.30 Qingquan Road, Laishan District, Yantai, Shandong 264005, China; College of Life Sciences, Yantai University, No.30 Qingquan Road, Laishan District, Yantai, Shandong 264005, China; College of Life Sciences, Yantai University, No.30 Qingquan Road, Laishan District, Yantai, Shandong 264005, China; College of Life Sciences, Yantai University, No.30 Qingquan Road, Laishan District, Yantai, Shandong 264005, China; College of Life Sciences, Yantai University, No.30 Qingquan Road, Laishan District, Yantai, Shandong 264005, China; College of Life Sciences, Yantai University, No.30 Qingquan Road, Laishan District, Yantai, Shandong 264005, China; College of Life Sciences, Yantai University, No.30 Qingquan Road, Laishan District, Yantai, Shandong 264005, China; College of Life Sciences, Yantai University, No.30 Qingquan Road, Laishan District, Yantai, Shandong 264005, China; College of Life Sciences, Yantai University, No.30 Qingquan Road, Laishan District, Yantai, Shandong 264005, China; College of Life Sciences, Yantai University, No.30 Qingquan Road, Laishan District, Yantai, Shandong 264005, China

## Abstract

Mollusca is the largest marine phylum, comprising about 23% of all named marine organisms, Mollusca systematics are still in flux, and an increase in human activities has affected Molluscan reproduction and development, strongly impacting diversity and classification. Therefore, it is necessary to explore the mitochondrial genome of Mollusca. The Mollusca mitochondrial database (MODB) was established for the Life and Health Big Data Center of Yantai University. This database is dedicated to collecting, sorting and sharing basic information regarding mollusks, especially their mitochondrial genome information. We also integrated a series of analysis and visualization tools, such as BLAST, MUSCLE, GENEWISE and LASTZ. In particular, a phylogenetic tree was implemented in this database to visualize the evolutionary relationships between species. The original version contains 616 species whose mitochondrial genomes have been sequenced. The database provides comprehensive information and analysis platform for researchers interested in understanding the biological characteristics of mollusks.

**Database URL**: http://modb.ytu.edu.cn/

## Introduction

Mollusca is the second-largest phylum of invertebrate animals after the Arthropoda; the number of valid recent species is currently estimated to be ∼110 000 and 23% of all named marine organisms are contained within this phylum (1, 2). Mollusks can adapt to different natural environments (3), from cold or temperate to tropical, and live in both freshwater and terrestrial habitats (4). Fossil shells recognizable as gastropods and bivalves are present in rocks from the Cambrian period, ∼ 570 million years ago (5). Mollusks vary in size from giant squids and clams to small snails measuring only a millimeter long. Despite their amazing diversity, all mollusks share some unique characteristics that define their body plan (6). The three most universal features defining modern mollusks are a mantle with a significant cavity used for breathing and excretion (7), the presence of a radula (except for bivalves) and the structure of the nervous system. In some groups, such as slugs and octopuses, the mantle is secondarily lost, while in others, it is used for other activities, such as respiration (8). Mollusks provide a clear example of a phenomenon called adaptive radiation (adaptation followed by spread in a particular niche). The gastropods and bivalves that were originally marine subsequently radiated into freshwater habitats (9). Without much change in gross appearance, these animals developed physiological mechanisms to retain salts within their cells and prevent excessive swelling from water intake in freshwater (10). Several groups of freshwater snails then produced species adapted to life on land. Gills adapted for the extraction of oxygen from water were transformed in land snails to lungs that extract oxygen from air, and the ammonia excretion typical of aquatic mollusks became uric acid excretion typical of birds and reptiles (11). Mollusks are of general importance within food chains and ecosystems, and certain species are of direct or indirect commercial and even medical importance to humans. Mitochondrial genes are preferred for the high copy number per cell, making them more likely to be collected than single-copy nuclear genes (12). Mitochondrial genes are inherited maternally and have a higher mutation rate than nuclear DNA. Because mitochondrial DNA (*mt*DNA) does not undergo recombination, *mt*DNA can be used to study genetic relationships between individuals in a population. Mitochondrial DNA can be used for species identification when morphology is unable to determine species and is also often used in the design of gene capture arrays (13).

Phylogenetic analyses based on mitochondrial gene sequences are usually restricted to closely related species due to the high rate of nucleotide substitutions. However, variations in mitochondrial gene content and sequences have been used to elucidate evolutionary relationships between abruptly related species based on commonly derived features indicating the common ancestor of a given population (14). Mollusca systematics are still in flux (15). There is still no agreement on some of the major relationships, and an increase in human activities has affected Molluscan reproduction and development (16), which strongly impacts diversity and classification. Fortunately, recent phylogenetic analyses based on multi-gene datasets have rendered promising results (17). In this regard, mitochondrial genomes have been widely used to reconstruct deep phylogenies (18). Therefore, it is necessary to explore the mitochondrial genome secrets of Mollusca.

Over the past decade or so, a number of mitochondrial databases have emerged with various functions. Researchers will choose databases with different data types and functions depending on their research needs and sometimes need to operate in more than one database. Some integrate species-specific mitochondrial genomes, such as HmtDB (https://www.hmtdb.uniba.it), an online database of annotated human mitochondrial genome sequences, which includes population data, and nucleotide and amino acid variability data (19). There are some databases that integrate mitochondrial data analysis tools, like MToolbox (https://sourceforge.net/projects/mtoolbox), a highly automated bioinformatic pipeline to reconstruct and analyze mitochondrial DNA from high throughput sequencing data (20). There are a number of databases that contain more than just somatic genomes and still have simple analytical functions, such as MitoAge (http://www.mitoage.info/), a database containing *mt*DNA data integrated with longevity records, which also includes tools for analysis of *mt*DNA with a focus on this in relation to longevity (21). This tool may be of use in the research of longevity.

For the phylum Mollusca, many databases have been developed to collect information on Mollusca. MolluscaBase (https://www.molluscabase.org/) aims to provide an authoritative, permanently updated account of all Molluscan species (22), while NMiTA (https://nmita.rsmas.miami.edu) aims to introduce Molluscan life habits. The databases mentioned above are related to the classification of Mollusca and their distribution. There are databases that focus on the integration and the use of genomic data from Mollusca. MolluscDB (http://mgbase.qnlm.ac) integrates genome-wide and transcriptome data from the Mollusca phylum to provide a clear view of genomic and transcriptomic data (23). Here, we unveil the Mollusca mitochondrial database (MODB), a public database dedicated to gathering, storing, analyzing and visualizing mitochondrial genomic datasets for Mollusca. In this database, we collected and stored 616 mitochondrial genomes of 616 species belonging to seven classes. All genomic information in MODB will be shared online and updated periodically according to new releases in both public databases and our laboratory. As a highly integrated information platform, the MODB provides online analysis tools. We believe that the web-based platform has a user-friendly interface and useful functions, which will undoubtedly contribute to extensive research in the field of ecology and on the biological characteristics of Mollusca.

### Overview of database structure and function

#### Database implementation

The MODB is implemented in a Linux operating system. The database was developed based on Scala 2.12.2 (https://www.scala-lang.org/), SBT 0.13.17 (https://www.scala-sbt.org/), Akka 2.12 (network server) (https://www.akka-technologies.com/) and MySQL 5.7.26 (database server) (https://www.mysql.com/). The interface components of the website were designed and implemented using Play Framework 2.6.25 (https://www.playframework.com/) and Bootstrap 3.3.0 (https://getbootstrap.com/), and JBrowser 1.12.3 (24) and Highmaps 6.1.0 (https://www.highcharts.com/) were used to realize genome browsing and geographical distribution.

#### Data collection and organization

**Figure 1.**
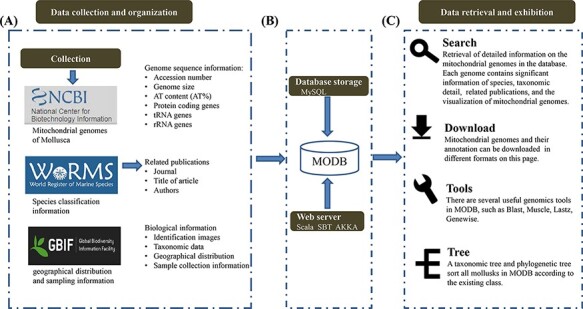
Schematic diagram of data processing for the MODB. (A) Data collection and data organization. (B) Building data association and adding data indexing and storage in a MySQL database. (C) Overview of the web interface and usage of MODB.

We collected and integrated the species information of 616 mollusks from the World Register of Marine Species (WoRMS, http://www.marinespecies.org/), Global Biodiversity Information Facility (GBIF, https://www.gbif.org/), National Center for Biotechnology Information (NCBI, https://www.ncbi.nlm.nih.gov/) and published literature (Figure 1). This database is dedicated to collecting, sorting and sharing basic information regarding mollusks, especially their mitochondrial gene information. At present, there are 616 species with mitochondrial genome sequencing in the MODB. Every mitochondrial sequence was obtained from NCBI and scanned by a self-written script. If the same data are found elsewhere, the program will give priority to retaining the data numbered ‘NC’. The 616 retained species were sorted and summarized, and the modification records were retained.

The data display interface contains the basic and sequence information for each species. This basic information includes species classification, geographical distribution and sampling information (GPS); sequence information includes mitochondrial length (length), AT content (AT%), genome information (site information, and positive and negative chain distribution) and literature sources.

#### Database home page

**Figure 2.**
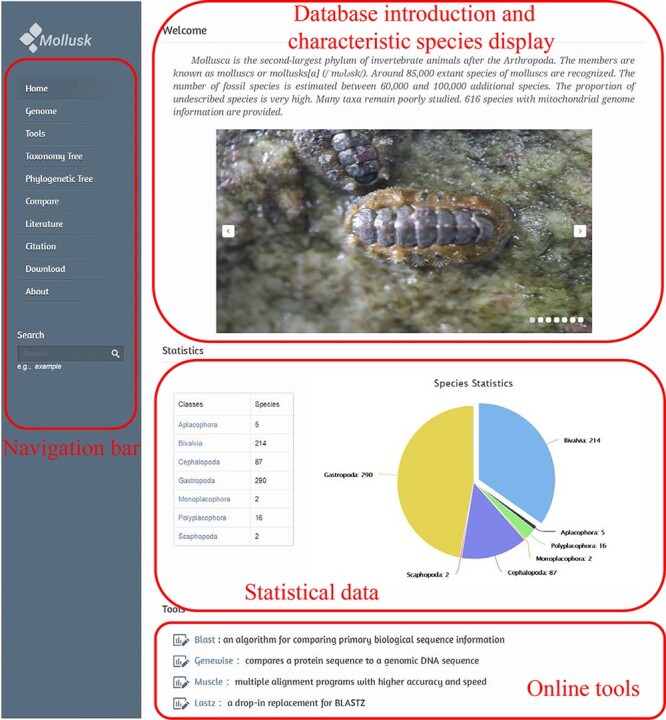
MODB home page.

The first page of the database consists of two parts: the navigation bar is on the left and the main content of the first page is on the right. The left navigation bar contains 11 tags, a search box and a database logo. Each of the tags represents a major function of the database (Figure 2).

#### Search and browse

**Figure 3.**
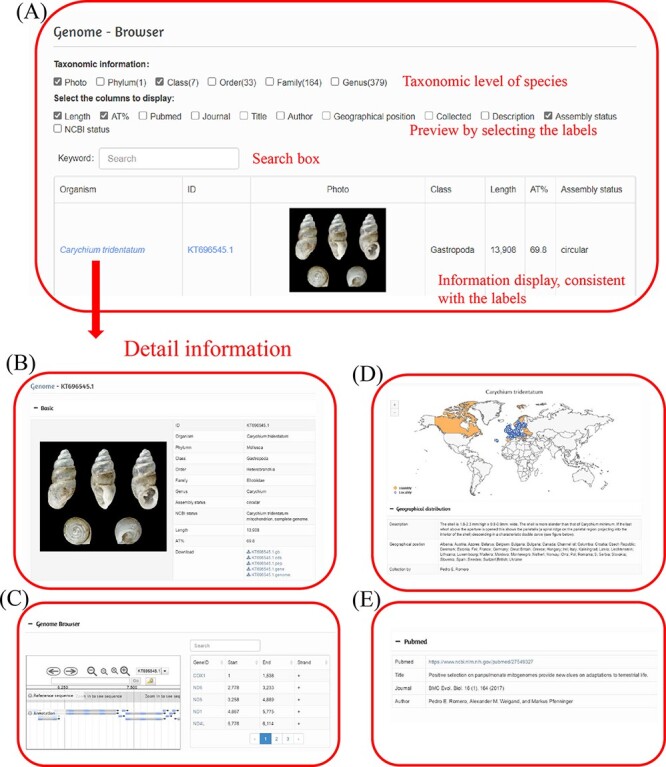
Data browsing and searching interface of the MODB. (A) Example of preview results, with the following detailed genome information: (B) Basic information on the mitochondrial genome; (C) Visual genome browser and coding genes of the mitochondrial genome; (D) Species distribution and sampling points; (E) Publications in which the generation of this record is described.

On the genome page, you can learn the classification information of all species in this database and the basic information of the mitochondrial genome. Users can check different display contents according to their own needs, preview this content and click the species of interest to view the specific information. You can also search for the target species directly in the search box. To assist users in quickly finding the data of interest, a smart search system was designed. Users can search the mitochondrial genomes of the desired species through a variety of different ways: by taxonomic level, scientific name and accession number. Users could enter one or more characters to select relevant data according to their own academic purposes (Figure 3A).

Each species page includes basic information, mitochondrial genome information, species distribution, sampling area and sequence source literature. The basic information includes taxon, sequence number, assembly effect, mitochondrial length and AT content (Figure 3B). The data were obtained from NCBI for this sequence sample, and the taxonomic information was compared and corrected with the World Register of Marine Species (WoRMS, http://www.marinespecies.org/). Furthermore, the sequence can be downloaded. The basic information on the mitochondrial genome is visualized through a genome browser, and gene information in the mitochondrial genome is displayed in table form (Figure 3C). The species distribution and sampling points are visualized in the form of a map, in which the species distribution is represented by country and marked with orange, while the sampling points are marked in blue (Figure 3D). The data on species distribution and sampling sites were obtained from the Global Biodiversity Information Facility (GBIF, https://www.gbif.org/). Sequence source literature is presented under the PubMed subheading at the bottom of the page (Figure 3E). A hyperlink connecting to the main body of the article is presented (using either PubMed or DOI number), and article title, journal and author information is also presented when available.

#### Phylogenetic tree of Mollusca

**Figure 4.**
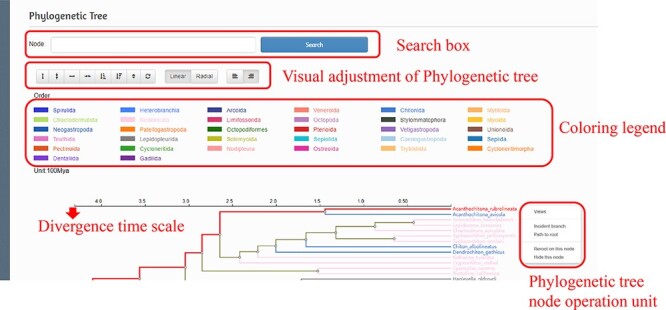
Phylogenetic tree interface and operation instructions.

To show the classification and evolution of Mollusca more clearly and intuitively, we used RAxML (25) to construct a phylogenetic tree containing 616 species based on concatenated sequences of all mitochondrial genomes and defined the divergence time. Within the interface, users can zoom in and out on the tree using the mouse wheel according to the user’s needs, and the bifurcation time will always be consistent with the phylogenetic tree so as to ensure the estimation of the bifurcation time of a node in the tree. In addition to the basic functions, the interface also includes the following functions: the species can be searched through the node search box, and the bifurcation time can be estimated according to the scale. Once the target species is selected, users can jump to the species details page, show branches, show divergent paths and hide nodes (Figure 4).

#### Sequence and information comparison of Mollusca

**Figure 5.**
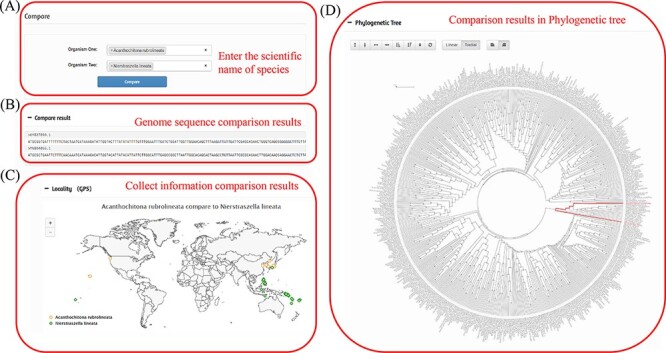
Comparison of two species in the MODB. (A) Input box for entering the scientific name of the species. (B) Comparative results of genome sequences. (C) Comparative results of species collection information. (D) Comparative results of evolutionary status.

Species information comparison is mainly conducted through the comparison tag. Users can input two organism names for comparison according to their own academic purposes. By inputting the species names of the two species (Figure 5A), the species information in the database can be compared, including sequence (Figure 5B), sampling point (Figure 5C) and status in the phylogenetic tree (Figure 5D). The basic information is mainly length comparison. The sampling points primarily reflect the different distributions of the two species and the different differentiation paths of the two species in the phylogenetic tree.

#### Online tools

There are several useful genomics tools in MODB that can aid researchers in exploring and analyzing the data, such as Blast (26), Muscle (27), Genewise (28) and Lastz (29). Users can upload their own data or use the data in the MODB database, fill in the parameters and submit the task. Then, a result page will appear automatically. The resulting file can be downloaded. However, Genewise only allows text input.

## Conclusion

We have developed the MODB database, which is a user-friendly data platform for Mollusca. MODB stores the biological information, genome sequence and gene information for 616 species of Mollusca in seven classes and is the most comprehensive database for integrating mitochondrial genomic resources in the phylum Mollusca. A phylogenetic tree of 616 species of the Mollusca phylum has been constructed, which is the largest phylogenetic tree based on the mitochondrial genomes of the Mollusca phylum. As MODB is a manually integrated database, all raw data collected and submitted will be reprocessed by our own custom pipeline, which will compare with near-source species and calibrate annotation information, including protein-coding genes, transfer RNA genes, ribosomal RNA genes, open reading frames and Introns. For this reason, different results may be produced when compared to the original published papers. The processed data are further associated with the retrieval and analysis modules. The retrieval and analysis modules, including various query, visualization and analysis tools, perform the main functions of the MODB database. We believe that the database can broaden the understanding of Mollusca’s basic knowledge and evolutionary relationships and attract more attention to the protection of the environment and Mollusca specifically. All genomic information in MODB will be shared online and regularly updated with new releases from public databases and our laboratory. Subsequently, we will continue to integrate, add mitochondrial genomic information of new species, further refine the nodes of the phylogenetic tree, increase divergence times and will integrate additional bioinformatic analysis tools to make MODB a more complete platform for sharing Molluscan information to achieve a comprehensive database for the integration and use of mitochondrial genomic resources.

## Limitations of the study

In this work, we scanned and re-annotated all collected mitochondrial genomes of mollusks to ensure the accuracy of the data. The database is currently in the initial operational capability phase, v1.0, and data sets as of December 2020, and the database will receive 2–3 major updates per year. At present, we only integrated the data in the NCBI-nucleotide database. As there are few sequencing data of some mollusk groups, there are many separate branches in the phylogenetic tree. Therefore, in the future, we will devote ourselves to integrating the mitochondrial genome data of mollusks in more public databases, and simultaneously, our research team will conduct more mollusk sequencing projects to provide more genomic data for mollusks.

## Data Availability

The MODB database can be accessed through the web server at http://modb.ytu.edu.cn/.
